# Differential impact of smoking on cardiac or non-cardiac death according to age

**DOI:** 10.1371/journal.pone.0224486

**Published:** 2019-10-30

**Authors:** Wonsuk Choi, Sun-Hwa Kim, Si-Hyuck Kang, Jin Joo Park, Chang-Hwan Yoon, Tae-Jin Youn, In-Ho Chae

**Affiliations:** Cardiovascular Center, Seoul National University Bundang Hospital, Seongnam, South Korea; International University of Health and Welfare, School of Medicine, JAPAN

## Abstract

Tobacco smoking causes cardiovascular diseases, lung disease, and various cancers. Understanding the population-based characteristics associated with smoking and the cause of death is important to improve survival. This study sought to evaluate the differential impact of smoking on cardiac or non-cardiac death according to age. Data from 514,866 healthy adults who underwent national health screening in South Korea were analyzed. The participants were divided into three groups: never-smoker, ex-smoker or current smoker according to the smoking status. The incidence rates and hazard ratios (HRs) of cardiac or non-cardiac deaths according to smoking status and age groups during the 10-year follow-up were calculated to evaluate the differential risk of smoking. Over the follow-up period, 6,192 and 24,443 cardiac and non-cardiac deaths had occurred, respectively. The estimated incidence rate of cardiac and non-cardiac death gradually increased in older age groups and was higher in current smokers and ex-smokers than that in never-smokers among all age groups. After adjustment of covariates, the HRs for cardiac death of current smokers compared to never-smokers were the highest in individuals in their 40’s (1.82; 95% CI, 1.45–2.28); this gradually decreased to 0.96 (95% CI, 0.67–1.38) in individuals >80 years. In contrast, the HRs for non-cardiac death peaked in individuals in their 50’s, (HR 1.69, 95% CI 1.57–1.82) and was sustained in those >80 years (HR 1.40, 95% CI 1.17–1.69). Ex-smokers did not show elevated risk of cardiac death compared to never-smokers in any age group, whereas they showed significantly higher risk of non-cardiac death in their 60’s and 70’s (HR, 1.29; 95% CI, 1.19–1.39; HR 1.22, 95% CI, 1.12–1.32, respectively). Acute myocardial infarction and lung cancer showed patterns similar to those of cardiac and non-cardiac death, respectively. Smoking was associated with higher relative risk of cardiac death in the middle-aged group and non-cardiac death in the older age group. Ex-smokers in the older age group had elevated risk of non-cardiac death. To prevent early cardiac death and late non-cardiac death, smoking cessation should be emphasized as early as possible.

## Introduction

Smoking is a risk factor for chronic obstructive pulmonary disease, cardiovascular diseases, and various cancers, including lung cancer and upper airway tract cancers.[1, 2]

Since the historical Framingham study first demonstrated adverse effects of smoking on coronary heart disease, various studies have confirmed the detrimental effects of smoking on cardiovascular diseases, including acute myocardial infarction (MI), heart failure (HF), stroke, and sudden cardiac arrest.[3–6]

Smoking has a significant positive exposure-response relationship with cardiovascular diseases, and smoking cessation mitigates the risk. A large meta-analysis with 141 cohort studies demonstrated that higher dose of smoking further increased the risk of acute MI and stroke.[7] Mons et al. showed that the risk for cardiovascular mortality, acute coronary events, and stroke events decreased in ex-smokers.[8] Furthermore, smoking is not merely a clinical risk factor for acute MI. Smokers tend to experience MI at an earlier age than do non-smokers, and smoking is more prevalent in young MI patients.[9–12]

Positive dose-response relationship between tobacco smoking and non-cardiovascular disease is also well known. In a meta-analysis of 140 studies, a clear linear correlation was seen between the dose of smoking and the relative risk for various types of lung cancer.[13]

However, the epidemiologic characteristics of smoking according to age on cardiac and non-cardiac death in general population are not well-established. This study was carried out to evaluate the differential impact of smoking on cardiac or non-cardiac death according to age by analyzing a cohort data from National Health Insurance System of South Korea.

## Methods

### Data collection

Analysis was conducted using data from the National Health Insurance System-National Health Screening Cohort (NHIS-HEALS).[14] The National Health Insurance Service is the only insurance provider in South Korea and covers nearly the entire Korean population. Participants of the insurance system get standardized medical examination every two years, including detailed medical history questionnaires, height, weight, blood pressure measurements, and laboratory tests.

Among participants of general screening program in 2002 and 2003, 10% of them were randomly selected to form the cohort. The cohort consisted of 514,866 subjects who were ≥40-years old at the time of enrollment and received routine examinations between 2002 and 2013. The cohort data consisted of demographic data, socioeconomic status, results of medical history questionnaires, including smoking habits, history of hospital visits, and death records in the cohort by the end of 2013.

Subjects with a previous history of acute MI, stroke, or lung cancer 1 year before the medical examination, those without current smoking status, or those without accurate death records were excluded from the study. Parameters, including age, sex, body mass index (BMI), socioeconomic status, geographic location, underlying disease, medication history, and laboratory results were extracted and included in the analysis. Parameters including age, BMI, systolic blood pressure (SBP), diastolic blood pressure (DBP) and laboratory findings including total cholesterol, fasting blood glucose or hemoglobin were collected as continuous variables, while parameters such as sex, economic status, underlying diseases or current medication were collected as discrete form. Economic status was divided into 6 different groups which are consisted of five quintile groups among insurance beneficiaries and a special group who were covered by medical aid (Table 1).

**Table 1:** Baseline characteristics of patients according to their smoking habits.

	Total (n = 478248)	Never-smoker (n = 327819)	Ex-smoker (n = 43162)	Current smoker (n = 107267)	*p-value*
**Age (years)**	54.27±9.60	55.07±9.72	53.36±91.41	52.20±8.92	< .001
**Male sex**	257028 (53.7%)	114225 (34.8%)	41161(95.4%)	101642 (94.8%)	< .001
**BMI (kg/m**^**2**^**)**	23.99±2.98	24.05±3.01	24.26±2.76	23.69±2.95	< .001
**SBP (mmHg)**	127.08±17.84	126.76±18.16	128.38±16.71	127.56±17.27	< .001
**DBP (mmHg)**	79.33±11.47	78.82±11.53	80.68±11.06	80.34±11.35	< .001
**Economic status, n (%)**					< .001
**Covered by medical aid**	1549 (0.3%)	1107 (0.3%)	98 (0.2%)	344 (0.3%)	
**First quintile (lowest)**	75180 (15.7%)	55188 (16.8%)	4446 (10.3%)	15546 (14.5%)	
**Second quintile**	65576 (13.7%)	46963 (14.3%)	4396 (10.2%)	14217 (13.3%)	
**Third quintile**	75001 (15.7%)	50369 (15.4%)	5971 (13.8%)	18661 (17.4%)	
**Fourth quintile**	99116 (20.7%)	65833 (20.1%)	9327 (21.6%)	23956 (22.3%)	
**Fifth quintile**	161826 (33.8%)	108359 (33.1%)	18924 (43.8%)	34543 (32.2%)	
**Underlying disease (%)**					
**HTN**	98342 (20.6%)	74105 (22.6%)	8725 (20.2%)	15512 (14.5%)	< .001
**DM**	28382 (5.9%)	19761 (6.0%)	2685 (6.2%)	5936 (5.5%)	< .001
**Dyslipidemia**	74339 (15.5%)	54672 (16.7%)	7107 (16.5%)	12560 (11.7%)	< .001
**CKD**	1445 (0.3%)	1093 (0.3%)	136 (0.3%)	216 (0.2%)	< .001
**Laboratory findings**					
**Total cholesterol (mg/dL)**	199.76±37.78	200.30±37.91	199.38±36.80	198.26±37.71	< .001
**Fasting blood glucose (mg/dL)**	98.47±31.76	97.55±30.65	100.05±29.53	100.67±35.58	< .001
**Hemoglobin (g/dL)**	13.88±1.52	13.49±1.47	14.68±1.22	14.78±1.23	< .001
**AST**	26.60±17.34	25.62±15.59	28.07±18.34	28.97±21.28	< .001
**ALT**	25.40±20.26	23.74±18.62	29.06±22.98	29.00±23.05	< .001
**Medication (%)**					
**Warfarin**	482 (0.1%)	351 (0.1%)	64 (0.1%)	67 (0.1%)	< .001
**Aspirin**	19683 (4.1%)	14659 (4.5%)	1895 (4.4%)	3129 (2.9%)	< .001
**Clopidogrel**	917 (0.2%)	661 (0.2%)	117 (0.3%)	139 (0.1%)	< .001
**Ticlopidine**	551 (0.1%)	409 (0.1%)	55 (0.1%)	87 (0.1%)	0.001
**ACEi or ARB**	26158 (5.5%)	19015 (5.8%)	2792 (6.5%)	4351 (4.1%)	< .001
**Beta blocker**	25399 (5.3%)	19273 (5.9%)	2198 (5.1%)	3928 (3.7%)	< .001
**CCB**	29717 (6.2%)	22103 (6.7%)	2761 (6.4%)	4853 (4.5%)	< .001
**Statin**	11931 (2.5%)	8923 (2.7%)	1175 (2.7%)	1833 (1.7%)	< .001

BMI; body mass index, SBP; systolic blood pressure, DBP; diastolic blood pressure, HTN; hypertension, DM; diabetes mellitus, CKD; chronic kidney disease, AST; aspartate transaminase, ALT; alanine transaminase, ACEi; Angiotensin-converting enzyme inhibitor, ARB; Angiotensin receptor blocker, CCB; calcium channel blocker

The insurance eligibility database was connected to the death certificate records from the National Death Index to confirm the date and cause of death of the enrollee. The primary endpoints were incidence rates of cardiac and non-cardiac deaths. Deaths without identifiable causes were considered as cardiac deaths. The secondary endpoints included acute myocardial infarction (MI), ischemic stroke, sudden cardiac arrest, and lung cancer.

This study was approved by the Institutional Review Board of the Seoul National University, Bundang Hospital (IRB number: X-1902-522-901). The requirement of informed consent from the patients was waived owing to the anonymized data set by the National Health Insurance System (NHIS) and retrospective design of the study.

### Definitions

The comorbidities were defined as prior diagnosis of any of these conditions at the time of cohort enrollment. Hypertension (HTN) was defined as the presence of the International Statistical Classification of Diseases and Related Health Problems, Tenth Revision (ICD-10) codes: hypertension (I10), hypertensive heart disease (I11), hypertensive renal disease (I12), hypertensive heart and renal disease (I13), or secondary hypertension (I15). Diabetes mellitus (DM) was defined as fasting blood glucose >126 mg/dL, any history of anti-diabetic medication or insulin prescription or the presence of the ICD-10 codes: type 2 DM (E11), malnutrition-related diabetes mellitus (E12), other specified diabetes mellitus (E13) or unspecified diabetes mellitus (E14). Dyslipidemia was defined as presence of the ICD-10 code: Disorders of lipoprotein metabolism and other lipidemias (E78). Chronic kidney disease (CKD) was defined as the presence of the ICD-10 code: chronic kidney disease (N18).

The smoking status was recorded as ‘never-smoker,’ ‘ex-smoker,’ or ‘current smoker’ as reported by the patient on the medical questionnaire. Self-reported questionnaire did not require specific duration of smoking cessation for the inclusion to ex-smoker group.

Medication history was defined as the presence of prescription history of corresponding medication during 1 month prior to enrollment.

Cardiac death was defined as the specific cause of death according to the ICD-10 codes: I00-I99. Acute MI was defined as the presence of the following ICD-10 codes: acute myocardial infarction (I21) and certain current complications following acute myocardial infarction (I23). Ischemic stroke was defined as the presence of the ICD-10 code for cerebral infarction (I63). Sudden cardiac arrest was defined as the presence of the ICD-10 code for cardiac arrest (I46). Lung cancer was defined as the presence of the ICD-10 code for malignant neoplasm of bronchus and lung (C34).

### Statistical analyses

The baseline characteristics of the subjects are presented as frequencies and percentages for the categorical variables and as means with standard deviations for the continuous variables. The categorical variables were analyzed using either chi-square or Fisher’s exact tests. The continuous variables were compared using the Student’s *t*-test or analysis of variance (ANOVA). A Cox-proportional hazard model was constructed to evaluate the hazard ratio for each event in both the groups. A multivariate regression model was constructed with adjustment for age, sex, BMI, socioeconomic status, and underlying diseases, such as HTN, DM, CKD, and dyslipidemia. All reported p-values are two-tailed, and *p*-values smaller than a significance level of alpha = 0.05 were considered statistically significant.

The statistical analyses were performed using R statistical Software v3.5.1 (R Development Core team, 2018).

## Results

After a excluding participants without relevant follow-up data, 501,033 subjects remained in the cohort. Subjects with a previous history of acute MI, stroke, or lung cancer (3,417 subjects), without current smoking status (19,366 subjects), older than 90 years (1 subjects) or without accurate death record (1 subjects) were excluded from the study, and 478,248 patients were included in the study. The population included 327,891 (68.5%) never-smokers, 43,162 (9.0%) ex-smokers, and 107,267 (22.4%) current smokers. The mean age was 54.27 ± 9.60 years, and 53.7% of the subjects were men. The mean BMI was 23.99 ± 2.98 kg/m^2^, mean SBP was 127.08±17.84 mmHg, and mean DBP was 79.33±11.47 mmHg. HTN, DM, and dyslipidemia were observed in 20.6, 5.9, 15.5% of the cohort population, respectively (Table 1).

**Fig 1:**
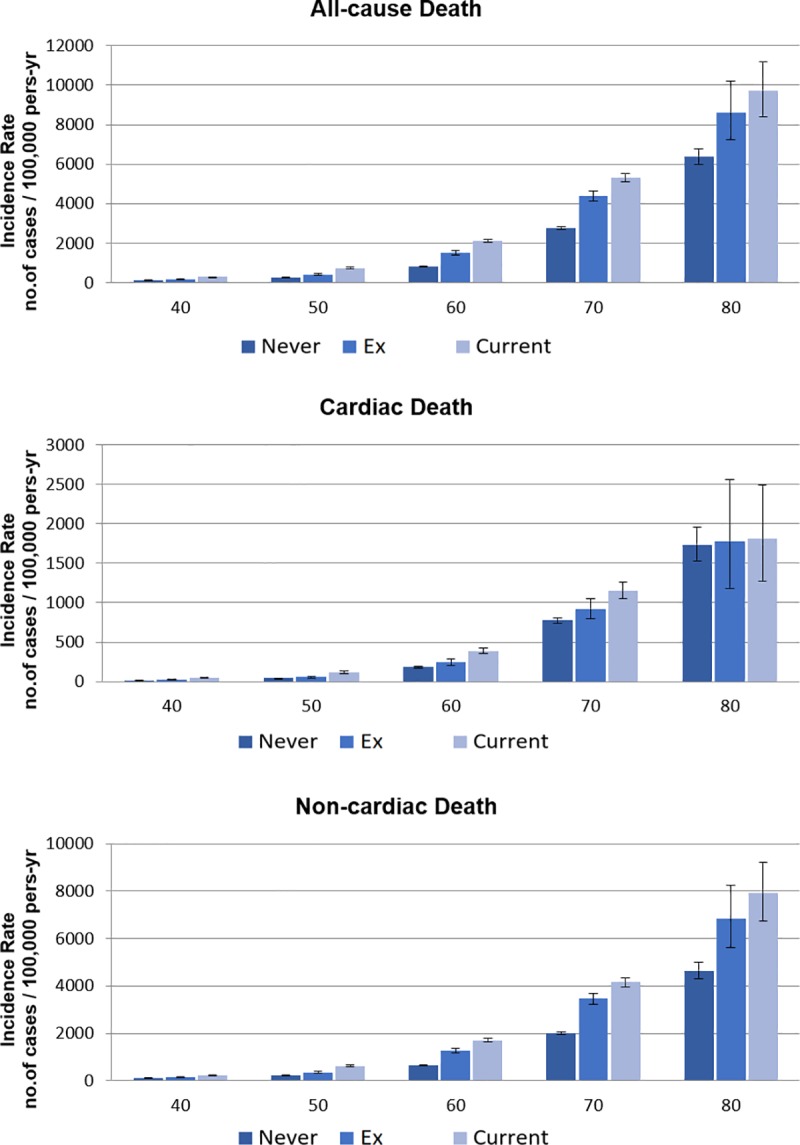
Estimated 100,000 person-year incidence rates of death according to their age and smoking habits.

**Fig 2:**
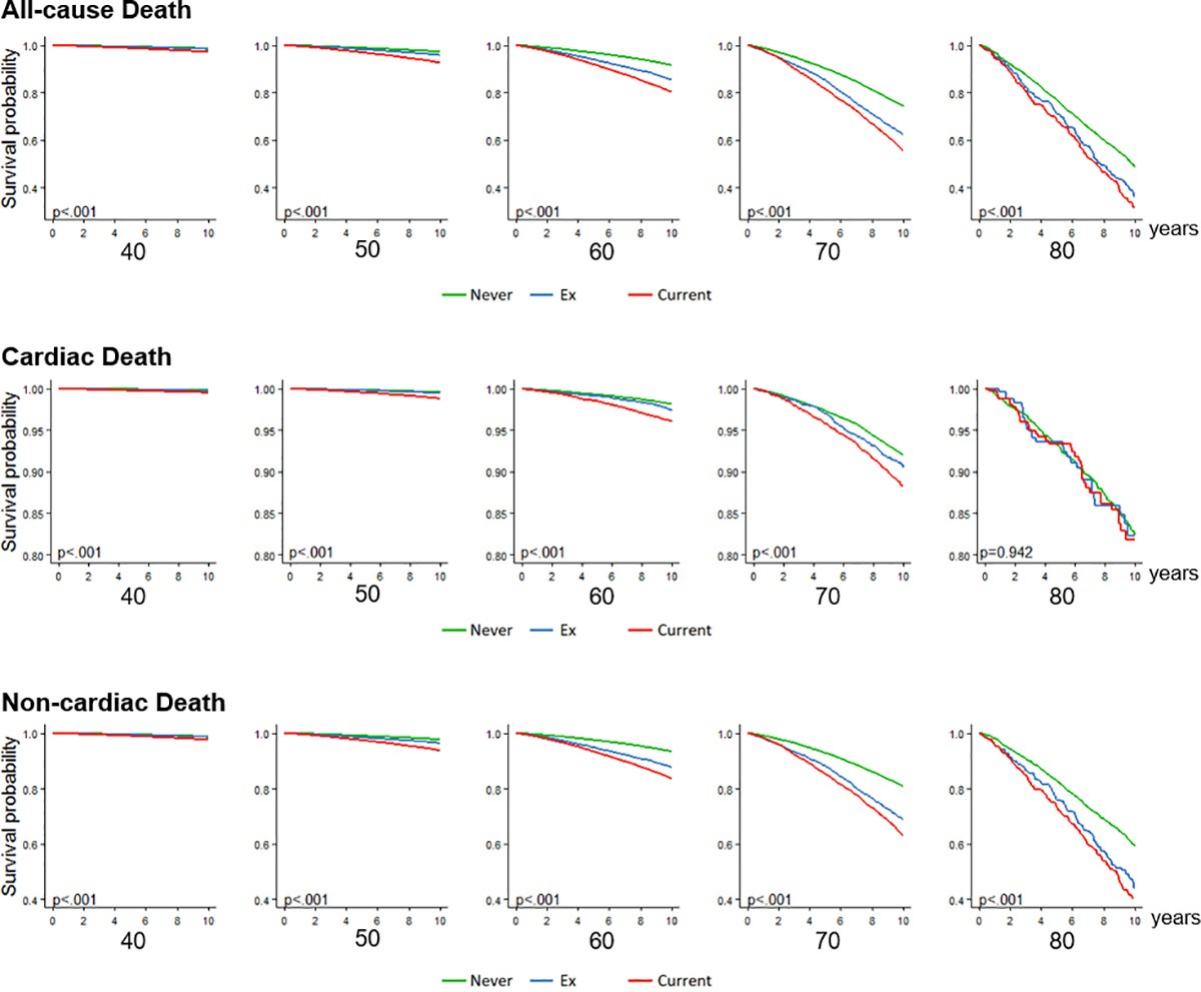
Kaplan-Meier 10-year cumulative survival curve according to their age and smoking habits.

After a median follow-up of 10 years, 6,192 and 24,443 cases of cardiac and non-cardiac death, respectively, had occurred. The estimated incidence rates of cardiac death were 134.65, 132.00, and 172.12 events per 100,000 person-year among never-smokers, ex-smokers, and current smokers, respectively. The rates of non-cardiac death among never-smokers, ex-smokers, and current smokers were 448.72, 622.27, and 766.06 per 100,000 person-year, respectively (Table 2). The incidence rates of cardiac and non-cardiac deaths gradually increased as the population became older, and current smokers or ex-smokers demonstrated a higher incidence of cardiac and non-cardiac deaths than do never-smokers in all age groups (Fig 1, Fig 2).

**Table 2:** Estimated 100,000 person-year incidence rates of all-cause death, cardiac death, and non-cardiac death according to their age and smoking status.

	Age (years)	Never-smoker	Ex-smoker	Current smoker
**All-cause death**	Total	583.37 (574.72–592.13)	754.27 (727.06–782.25)	938.18 (918.98–957.67)	
	40	127.44 (120.83–134.31)	159.65 (141.50–179.49)	283.31 (268.54–298.69)	
	50	226.77 (256.35–277.50)	407.52 (371.91–445.62)	740.54 (709.48–772.61)	
	60	835.75 (814.35–857.56)	1506.29 (1414.18–1602.82)	2100.5 (2027.66–2175.28)	
	70	2760.88 (2695.28–2827.67)	4372.29 (4105.99–4651.34)	5303.12 (5085.18–5528.00)	
	80	6364.40 (5965.45–6783.01)	8612.21 (7225.67–10187.28)	9718.23 (8413.96–11165.18)	
**Cardiac death**	Total	134.65 (130.51–138.89)	132.00 (120.76–144.01)	172.12 (163.95–180.59)	
	40	16.94 (14.58–19.56)	23.95 (17.26–32.37)	47.36 (41.43–53.89)	
	50	43.42 (39.28–47.88)	54.22 (41.26–69.24)	116.38 (104.27–129.51)	
	60	182.98 (173.04–193.34)	245.00 (208.72–285.77)	388.66 (357.69–421.59)	
	70	774.28 (739.72–810.03)	918.88 (799.07–1051.58)	1147.26 (1047.12–1254.41)	
	80	1733.91 (1528.77–1958.91)	1773.1 (1178.21–2526.63)	1806.72 (1272.10–2490.33)	
**Non-cardiac death**	Total	448.72 (441.13–456.40)	622.27 (597.57–647.73)	766.06 (748.72–783.70)	
	40	110.50 (104.35–116.91)	135.70 (119.01–154.08)	235.86 (222.49–250.02)	
	50	233.35 (213.83–233.19)	353.30 (320.20–388.89)	624.16 (595.67–653.66)	
	60	652.77 (633.88–672.08)	1261.29 (1177.13–1349.88)	1711.84 (1646.15–1779.48)	
	70	1986.60 (1931.02–2043.38)	3453.42 (3217.21–3702.38)	4155.86 (3963.19–4355.47)	
	80	4630.49 (4291.14–4989.53)	6839.11 (5610.25–8257.13)	7910.51 (6739.26–9226.80)	

After adjustment of covariates, the hazard ratio for the all-cause deaths among current smokers compared to never-smokers was 1.64 (95% CI 1.59 to 1.69, p<0.001). The ratio for ex-smokers was smaller than that for the current smokers (Hazard ratio = 1.15, 95% CI 1.11–1.20, p<0.001).

**Fig 3:**
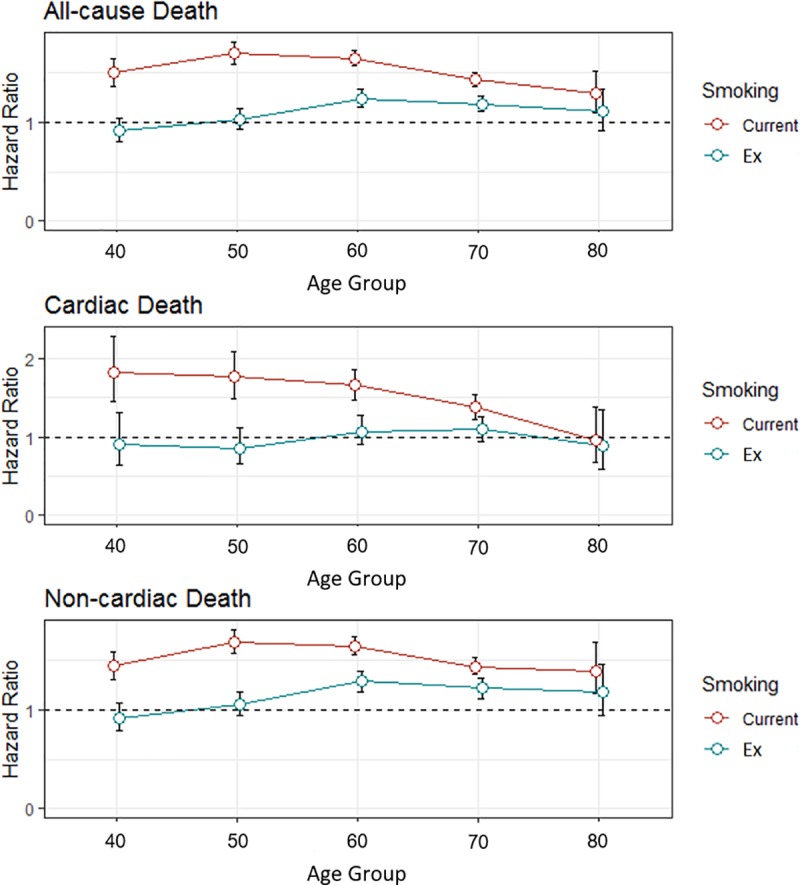
Adjusted hazard ratio and 95% confidence intervals of current smokers and ex-smokers compared to never-smokers in various outcomes.

The adjusted hazard ratio for cardiac death of current smokers was significantly higher than that for never-smokers (HR 1.65, 95% CI 1.55–1.77, p<0.001). The hazard ratio for cardiac death of current smokers versus never-smokers was the highest in their 40’s, (HR 1.82, 95% CI 1.45–2.28) and then gradually decreased to the level of insignificance in their 80’s (HR 0.96, 95% CI 0.67–1.38, p = 0.822) (Table 3, Fig 3).

**Table 3:** Hazard ratio of smoking on all-cause death, cardiac death, and non-cardiac death; univariate and multivariate analysis.

		Univariate analysis			Multivariate analysis		
	Age (years)	Smoking	HR^a^ (95% CI)	p-value	Smoking	HR^a^ (95% CI)	p-value
**All-cause death**	Total	Ex	1.29 (1.24–1.35)	< .001	Ex	1.15 (1.11–1.20)	< .001
		Current	1.61 (1.57–1.65)	< .001	Current	1.64 (1.59–1.69)	< .001
	40	Ex	1.25 (1.10–1.42)	0.001	Ex	0.92 (0.80–1.05)	0.216
		Current	2.22 (2.06–2.39)	< .001	Current	1.50 (1.37–1.64)	< .001
	50	Ex	1.53 (1.39–1.69)	< .001	Ex	1.03 (0.93–1.14)	0.610
		Current	2.78 (2.62–2.94)	< .001	Current	1.70 (1.59–1.82)	< .001
	60	Ex	1.82 (1.70–1.95)	< .001	Ex	1.24 (1.16–1.34)	< .001
		Current	2.53 (2.42–2.65)	< .001	Current	1.65 (1.57–1.73)	< .001
	70	Ex	1.61 (1.51–1.72)	< .001	Ex	1.19 (1.11–1.27)	< .001
		Current	1.96 (1.87–2.06)	< .001	Current	1.43 (1.36–1.51)	< .001
	80	Ex	1.37 (1.15–1.64)	0.001	Ex	1.11 (0.92–1.34)	0.287
		Current	1.55 (1.33–1.81)	< .001	Current	1.29 (1.10–1.52)	0.002
**Cardiac death**	Total	Ex	0.98 (0.89–1.08)	0.686	Ex	1.03 (0.93–1.14)	0.552
		Current	1.28 (1.21–1.35)	< .001	Current	1.65 (1.55–1.77)	< .001
	40	Ex	1.41 (1.01–1.98)	0.043	Ex	0.91 (0.64–1.30)	0.616
		Current	2.79 (2.30–3.39)	< .001	Current	1.82 (1.45–2.28)	< .001
	50	Ex	1.25 (0.96–1.63)	0.094	Ex	0.85 (0.65–1.12)	0.225
		Current	2.68 (2.32–3.10)	< .001	Current	1.76 (1.48–2.08)	< .001
	60	Ex	1.35 (1.15–1.59)	< .001	Ex	1.07 (0.90–1.27)	0.432
		Current	2.14 (1.94–2.36)	< .001	Current	1.65 (1.47–1.85)	< .001
	70	Ex	1.21 (1.05–1.39)	0.010	Ex	1.09 (0.94–1.26)	0.272
		Current	1.51 (1.37–1.67)	< .001	Current	1.37 (1.22–1.53)	< .001
	80	Ex	1.04 (0.70–1.53)	0.857	Ex	0.89 (0.59–1.35)	0.593
		Current	1.06 (0.75–1.49)	0.754	Current	0.96 (0.67–1.38)	0.822
**Non-cardiac death**	Total	Ex	1.39 (1.33–1.45)	< .001	Ex	1.18 (1.13–1.24)	< .001
		Current	1.70 (1.66–1.75)	< .001	Current	1.63 (1.58–1.68)	< .001
	40	Ex	1.23 (1.07–1.41)	0.004	Ex	0.92 (0.79–1.07)	0.264
		Current	2.13 (1.97–2.31)	< .001	Current	1.45 (1.31–1.59)	< .001
	50	Ex	1.59 (1.43–1.76)	< .001	Ex	1.06 (0.95–1.19)	0.291
		Current	2.80 (2.63–2.98)	< .001	Current	1.69 (1.57–1.82)	< .001
	60	Ex	1.95 (1.81–2.10)	< .001	Ex	1.29 (1.19–1.39)	< .001
		Current	2.64 (2.52–2.77)	< .001	Current	1.65 (1.56–1.74)	< .001
	70	Ex	1.77 (1.64–1.91)	< .001	Ex	1.22 (1.12–1.32)	< .001
		Current	2.14 (2.02–2.26)	< .001	Current	1.44 (1.36–1.53)	< .001
	80	Ex	1.50 (1.23–1.84)	< .001	Ex	1.18 (0.95–1.46)	0.127
		Current	1.74 (1.46–2.06)	< .001	Current	1.40 (1.17–1.69)	< .001

HR; hazard ratio, CI; confidence interval, Ex; ex-smoker, current; current smoker

^a^ Hazard ratio of various outcomes according to smoking status compared to never smoker.

In the case of non-cardiac death, the hazard ratio of current smokers versus never-smokers was 1.63 (95% CI 1.59–1.68, p < 0.001). The current smokers demonstrated consistently elevated risk of non-cardiac death compared to never-smokers in all age groups (all, p < 0.001). The risk for non-cardiac death peaked in their 50’s (HR 1.69, 95% CI 1.57–1.82, p<0.001) and 60’s (HR 1.65, 95% CI 1.56–1.74, p<0.001), and maintained its significance until their 80’s (HR 1.40, 95% CI 1.17–1.69, p<0.001) (Table 3, Fig 3).

Interestingly, ex-smokers did not show higher risk for cardiac death than did never-smokers (HR 1.03, 95% CI 0.93–1.14, p = 0.552). The unadjusted risk of cardiac death for ex-smokers was elevated in their 40’s, 60’s, and 70’s, but it was not significant after multivariate analysis. The result was consistent among all age groups (all, p > 0.05, Table 3).

On the contrary, the hazard ratio for non-cardiac death among ex-smokers versus never-smokers was slightly elevated (HR 1.18, 95% CI 1.13–1.24, p < 0.001). They had a significant elevated risk for non-cardiac death in their 60’s (HR 1.29, 95% CI 1.19–1.39, p<0.001) and 70’s (HR 1.22, 95% CI 1.12–1.32, p<0.001) (Fig 3).

Cardiovascular diseases, including acute MI, ischemic stroke, and sudden cardiac arrest demonstrated similar pattern with cardiac death. Their incidence rates increased when the cohort population became older, and current smoker and ex-smoker in the same age group showed a tendency for higher incidence of these diseases than did never-smokers (S1 Table, S1 Fig). The adjusted hazard ratio for acute MI, ischemic stroke, and sudden cardiac death among current smokers versus never-smokers was highest in their 40’s and 50’s, and continuously decreased as the population aged. That hazard ratio of ex-smokers compared to never- smokers was not elevated in general (S2 Table, S2 Fig).

Meanwhile, lung cancer demonstrated similar increase in incidence rate based on the age of the population or the smoking status with non-cardiac death (S1 Table, S1 Fig). The hazard ratio for lung cancer of current smokers versus never-smokers peaked in their 60’s and 70’s, which was similar to the pattern for non-cardiac death. The hazard ratio among ex-smokers compared to never-smokers was smaller than that among current smokers, especially in their 60’s and 70’s (HR 1.28, 95% CI = 1.14–1.43, p < 0.001, and HR 1.24, 95% CI 1.08–1.42, p = 0.003, respectively, S2 Table, S2 Fig).

## Discussion

In this study, we found that the hazard ratio for cardiac death or various cardiovascular diseases in smokers compared to never-smokers was highest in the middle-aged group and continuously decreased with age, while the risk for non-cardiac death or lung cancer consistently increased throughout their life. The hazard ratio of current smokers versus never-smokers in their 40’s was 1.8 for cardiac death, while it decreased to a statistically insignificant value for those in their 80’s. However, a decrease in the hazard of active smoking according to age was not observed in non-cardiac death. The hazard ratio of current smokers compared to never-smokers for non-cardiac death was 1.45 for subjects in their 40’s and remained significantly high, regardless of the age of cohort population.

Several previous studies have reported elevated risk of various cardiovascular diseases, especially that in young smokers.[4–7, 9, 12, 15, 16] In one study that reported the risk of smoking in patients with ST-elevation myocardial infarction (STEMI) according to age, the incidence of STEMI was 8.5 times higher in younger smokers, while that was only 3.1 times higher in smokers who were older than 65 years.[17] However, previous studies were mainly cross-sectional or observational studies which involves relatively small number of patients.[12, 15, 16] Even in large cohort studies and meta-analyses, they usually focused on one or two diseases.[4, 5, 8] On the contrary, our study is a large cohort study which encompasses more than 500,000 participants over 10 years, and we investigated multiple cause of death and various diseases including cardiac death, non-cardiac death, acute myocardial infarction, sudden cardiac death, ischemic stroke and lung cancer. In the process we could discover general overview of harmful effects of smoking and benefits of smoking cessation on diverse disease entities. We could also identify the detailed magnitude of hazard ratio of active smoking or former smoking on several diseases according to the patients’ age.

It is difficult to explain the decreased magnitude of cardiovascular risk of active smoking especially in the older generation with our data. One possible mechanism is that younger smokers tend to have lesser cardiovascular risk factors, such as hypertension, diabetes, or dyslipidemia. Although we observed the phenomenon even after adjustment with multiple covariates, unmeasured confounding factors could exist. Another possible explanation is that smoking causes acute vessel wall inflammation and injury, and early cardiovascular events can occur in a susceptible population. Smoking causes inflammation, thrombosis, oxidation of low-density lipoprotein cholesterol, and increases oxidative stress.[18] A study conducted with *in vivo* virtual histology intravascular ultrasound showed more necrotic cores in the coronary plaques among smokers than those among non-smokers.[19] Genetic predisposition could play a role in the early development of atherosclerosis in susceptible smokers. CYP1A1 *Msp* polymorphism was associated with multi-vessel coronary artery disease in light smokers.[20] In another study, smoking reduced the activity of endothelial nitric oxide synthase in a specific allele.[21] Hence, it is possible that a population relatively resistant to acute vascular injury could remain in the cohort leading to the decreased hazard of smoking in older population, so called, a survivorship bias. In our cohort data, the proportion of current smokers continuously decreased among older subjects, while that of ex-smokers was relatively constant (S3 Fig). It could be explained by survivorship bias. Subjects who were genetically susceptible to adverse effects of the various harmful substances found in cigarette smoking would suffer from early death caused by a smoking-related disease, while subjects who were resistant survived and accounted for a larger proportion among the older smokers. However, this survivorship bias hypothesis is not well-validated and further researches are needed. More and more people seemed to choose to quit smoking as they got older. Notably, the proportion of ex-smokers to current smoker was higher in the older age groups.

Finally, increased incidence of non-cardiac death among current smokers could reduce the possibility of an affected subject dying of cardiovascular diseases, as the primary end points, such as cardiac death or non-cardiac death are mutually exclusive. In our study, smoking cessation in the general population certainly eliminated the smoking-associated risk of cardiac death and cardiovascular disease. It is concordant with previous studies in which the risk for cardiovascular disease was at least partially reversible when patients quit smoking, although it needed a certain time period.[1, 7, 10, 17, 22, 23] The excessive risk of heart attack caused by smoking reduces by 50% within 1 year of smoking cessation.[1] Another study demonstrated that after 15 years of smoking cessation, the risk of MI in ex-smokers was not different from that in never-smokers.[22] A meta-analysis of prospective cohort studies showed that smoking cessation reduces mortality even when smoking cessation occurred after the diagnosis of coronary artery disease.[24]

However, we could not obtain accurate duration of smoking cessation on ex-smoker group. Therefore, participants who are in their earliest stages of smoking cessation could be included in the ex-smoker group. This obscure classification of ex-smoker group could have possibly influenced worse outcome of ex-smoker group in the study. Unfortunately, various smoking cessation methods demonstrated various success rates and many smokers are still suffering on their attempt to quit smoking. [1, 25] In fact, length of smoking cessation was linearly correlated with the improvement on prognosis of various cardiovascular diseases including sudden cardiac death and acute coronary heart disease. [22, 26]

On the contrary, elevated risks for non-cardiac death in both smokers and ex-smokers were continuously observed, especially in the older age group. Negative impact of smoking on pulmonary function was often irreversible, and harmful effects of smoking on pulmonary function and risk for cancers have not been fully recovered, despite smoking cessation.[1] In lung cancer, the exposure-response relationship between smoking and risk was almost linear, unlike cardiovascular diseases, which showed a morbid risk starting from low-exposure levels.[27] It suggests that the mechanisms of smoking hazards on cardiac death and non-cardiac death are different. For example, cancer induction involves carcinogen exposure, metabolic activation, DNA adduct formation, and consequent mutation of critical genes along with the exacerbating influences of inflammation, co-carcinogenesis, and tumor promotion.[28] The average mutation frequency is reported to be more than 10-fold higher in smokers than that in never-smokers.[29] Therefore, detrimental effect of smoking on non-cardiac death or lung cancer cannot be reversed by smoking cessation and persists in the older age cohort.

This cohort data lacks the information on the dose of smoking. However, 80% of Korean smokers, including ex-smokers smoke more than half a pack a day over 10 years.[30] As even light smokers have significant cardiovascular and cancer risks, the findings of this study may not be affected by the dose and duration of the smoking.

## Conclusion

Smoking was associated with higher relative risks of cardiac death in the middle-aged group and, non-cardiac death throughout their adulthood period, when compared to never-smokers. On the contrary, elevated hazard ratio in ex-smokers versus non-smokers was only prominent for non-cardiac death in the older age group. To prevent early cardiac death and non-cardiac death throughout their life, smoking cessation should be emphasized among the younger population as early as possible.

## Supporting information

S1 Table Estimated 100,000 person-year incidence rates of acute MI, ischemic stroke, sudden cardiac arrest, and lung cancer according to their age and smoking status. (DOCX)

S2 Table Hazard ratio of smoking on other endpoints; univariate and multivariate analysis. (DOCX)

S1 Fig Estimated 100,000 person-year incidence rates of cardiovascular diseases according to their age and smoking habits. (DOCX)

S2 Fig Adjusted hazard ratio and 95% confidence intervals of current smokers and ex-smokers in various outcomes. (DOCX)

S3 Fig Proportion of current smokers according to age. (DOCX)
