# Supplementary material for: Differential impact of smoking on cardiac or non-cardiac death according to age
Source: PLoS One. 2019 Oct 30;14(10):e0224486. doi: 10.1371/journal.pone.0224486 (PMC6821404; doi:10.1371/journal.pone.0224486)
Supplement: S1 Table — (DOCX) [file pone.0224486.s001.docx]

**S1 Table. Estimated 100,000 person-year incidence rates of acute MI, ischemic stroke, sudden cardiac arrest, and lung cancer according to their age and smoking status**

|  | **Age (years)** | **Never-smoker** | **Ex-smoker** | **Current smoker** |
| --- | --- | --- | --- | --- |
| **Acute MI** | Total | 89.36 (85.99-92.83) | 118.76 (108.09-130.20) | 175.24 (166.97-183.82) |
|  | 40 | 32.46 (29.16-36.02) | 58.31 (47.55-70.79) | 111.7 (102.47-121.53) |
|  | 50 | 63.90 (58.85-69.27) | 108.12 (90.14-128.65) | 183.59 (168.23-199.97) |
|  | 60 | 138.96 (130.3-148.05) | 199.64 (166.92-236.91) | 283.57 (257.10-312.04) |
|  | 70 | 283.34 (262.5-305.39) | 365.22 (290.9-452.74) | 461.19 (398.10-531.43) |
|  | 80 | 400.26 (304.70-516.31) | 704.86 (351.86-1261.18) | 392.71 (169.54-773.79) |
| **Ischemic stroke** | Total | 239.67 (234.11-245.33) | 225.30 (210.47-240.89) | 275.50 (265.09-286.21) |
|  | 40 | 50.93 (46.78-55.35) | 79.54 (66.87-93.92) | 104.02 (95.13-113.52) |
|  | 50 | 146.31 (138.60-154.34) | 150.09 (128.73-173.97) | 267.07 (248.45-286.71) |
|  | 60 | 423.73 (408.41-439.47) | 480.98 (429.09-537.42) | 617.75 (578.13-659.37) |
|  | 70 | 880.22 (842.83-918.84) | 941.24 (818.51-1077.17) | 1091.37 (992.2-1197.76) |
|  | 80 | 1249.69 (1073.79-1446.18) | 1309.48 (799.86-2022.38) | 1297.16 (847.34-1900.63) |
| **Sudden cardiac arrest** | Total | 19.27 (17.72-20.91) | 23.17 (18.61-28.52) | 36.27 (32.57-40.27) |
|  | 40 | 6.23 (4.83-7.89) | 7.41 (3.95-12.68) | 18.94 (15.27-23.23) |
|  | 50 | 12.1 (9.96-14.56) | 13.56 (7.75-22.02) | 36.03 (29.44-43.65) |
|  | 60 | 28.45 (24.62-32.72) | 57.48 (48.67-78.89) | 68.24 (55.64-82.84) |
|  | 70 | 72.67 (62.36-84.20) | 82.75 (49.82-129.22) | 114.26 (84.24-151.49) |
|  | 80 | 120.97 (71.70-191.19) | 189.99 (39.18-555.22) | 244.16 (79.28-569.78) |
| **Lung cancer** | Total | 216.77 (211.48-222.15) | 293.22 (276.27-310.95) | 392.76 (380.31-405.52) |
|  | 40 | 88.19 (82.70-93.95) | 104.78 (90.14-121.10) | 139.28 (128.95-150.21) |
|  | 50 | 168.05 (159.78-176.64) | 222.05 (195.88-250.75) | 370.43 (348.44-393.44) |
|  | 60 | 328.49 (315.06-342.35) | 596.85 (538.93-659.29) | 907.90 (859.73-958.05) |
|  | 70 | 625.15 (593.87-657.65) | 1170.08 (1032.42-1320.98) | 1649.21 (1527.04-1778.56) |
|  | 80 | 938.53 (787.96-1109.50) | 1565.84 (1003.26-2329.84) | 1447.80 (969.62-2079.29) |
|  |  |  |  |  |

HR; hazard ratio, CI; confidence interval, Ex; ex-smoker, current; current smoker
