# Supplementary material for: Differential impact of smoking on cardiac or non-cardiac death according to age
Source: PLoS One. 2019 Oct 30;14(10):e0224486. doi: 10.1371/journal.pone.0224486 (PMC6821404; doi:10.1371/journal.pone.0224486)
Supplement: S2 Table — (DOCX) [file pone.0224486.s002.docx]

**S2 Table. Hazard ratio of smoking on other endpoints; univariate and multivariate analysis**

|  |  | **Univariate analysis** | | | **Multivariate analysis** | | |
| --- | --- | --- | --- | --- | --- | --- | --- |
|  | **Age (years)** | **Smoking** | **HR (95% CI)** | **p-value** | **Smoking** | **HR (95% CI)** | **p-value** |
| **Acute MI** | Total | Ex | 1.33 (1.20-1.47) | <.001 | Ex | 1.08 (0.97-1.20) | 0.157 |
|  |  | Current | 1.96 (1.84-2.08) | <.001 | Current | 1.86 (1.73-2.00) | <.001 |
|  | 40 | Ex | 1.80 (1.44-2.24) | <.001 | Ex | 1.05 (0.83-1.32) | 0.694 |
|  |  | Current | 3.44 (3.01-3.93) | <.001 | Current | 2.09 (1.79-2.45) | <.001 |
|  | 50 | Ex | 1.69 (1.40-2.05) | <.001 | Ex | 1.08 (0.88-1.32) | 0.473 |
|  |  | Current | 2.87 (2.55-3.23) | <.001 | Current | 1.86 (1.63-2.14) | <.001 |
|  | 60 | Ex | 1.44 (1.20-1.73) | <.001 | Ex | 1.05 (0.87-1.28) | 0.597 |
|  |  | Current | 2.04 (1.82-2.29) | <.001 | Current | 1.63 (1.43-1.86) | <.001 |
|  | 70 | Ex | 1.29 (1.03-1.62) | 0.029 | Ex | 1.06 (0.83-1.35) | 0.645 |
|  |  | Current | 1.63 (1.39-1.91) | <.001 | Current | 1.48 (1.24-1.77) | <.001 |
|  | 80 | Ex | 1.74 (0.92-3.32) | 0.090 | Ex | 1.67 (0.83-3.35) | 0.153 |
|  |  | Current | 0.98 (0.47-2.04) | 0.948 | Current | 0.91 (0.42-1.97) | 0.818 |
| **Ischemic stroke** | Total | Ex | 0.94 (0.88-1.01) | 0.089 | Ex | 0.96 (0.89-1.03) | 0.238 |
|  |  | Current | 1.15 (1.10-1.20) | <.001 | Current | 1.40 (1.33-1.47) | <.001 |
|  | 40 | Ex | 1.56 (1.30-1.88) | <.001 | Ex | 1.21 (0.99-1.49) | 0.063 |
|  |  | Current | 2.04 (1.81-2.30) | <.001 | Current | 1.59 (1.37-1.84) | <.001 |
|  | 50 | Ex | 1.03 (0.88-1.20) | 0.743 | Ex | 0.81 (0.68-0.95) | 0.011 |
|  |  | Current | 1.83 (1.67-2.00) | <.001 | Current | 1.48 (1.33-1.64) | <.001 |
|  | 60 | Ex | 1.14 (1.01-1.28) | 0.027 | Ex | 0.99 (0.87-1.12) | 0.829 |
|  |  | Current | 1.46 (1.36-1.58) | <.001 | Current | 1.33 (1.22-1.45) | <.001 |
|  | 70 | Ex | 1.07 (0.93-1.24) | 0.321 | Ex | 0.95 (0.81-1.10) | 0.460 |
|  |  | Current | 1.25 (1.13-1.38) | <.001 | Current | 1.19 (1.07-1.33) | 0.002 |
|  | 80 | Ex | 1.05 (0.66-1.66) | 0.843 | Ex | 0.93 (0.57-1.51) | 0.776 |
|  |  | Current | 1.04 (0.69-1.57) | 0.856 | Current | 0.96 (0.63-1.48) | 0.867 |
| **Sudden cardiac arrest** | Total | Ex | 1.20 (0.96-1.51) | 0.102 | Ex | 0.95 (0.75-1.19) | 0.641 |
|  |  | Current | 1.87 (1.64-2.14) | <.001 | Current | 1.70 (1.46-1.97) | <.001 |
|  | 40 | Ex | 1.19 (0.66-2.15) | 0.571 | Ex | 0.68 (0.37-1.25) | 0.215 |
|  |  | Current | 3.03 (2.22-4.15) | <.001 | Current | 1.67 (1.17-2.38) | 0.005 |
|  | 50 | Ex | 1.13 (0.67-1.90) | 0.654 | Ex | 0.67 (0.39-1.15) | 0.143 |
|  |  | Current | 2.98 (2.28-3.89) | <.001 | Current | 1.72 (1.27-2.33) | <.001 |
|  | 60 | Ex | 2.06 (1.46-2.92) | <.001 | Ex | 1.32 (0.91-1.90) | 0.141 |
|  |  | Current | 2.43 (1.91-3.09) | <.001 | Current | 1.59 (1.21-2.08) | 0.001 |
|  | 70 | Ex | 1.17 (0.73-1.88) | 0.512 | Ex | 0.90 (0.55-1.48) | 0.691 |
|  |  | Current | 1.63 (1.18-2.24) | 0.003 | Current | 1.39 (0.98-1.96) | 0.064 |

**S2 Table. Hazard ratio of smoking on other endpoints; univariate and multivariate analysis (continued)**

|  |  | **Univariate analysis** | | | **Multivariate analysis** | | | |
| --- | --- | --- | --- | --- | --- | --- | --- | --- |
|  | **Age (years)** | **Smoking** | **HR (95% CI)** | **p-value** | **Smoking** | **HR (95% CI)** | | **p-value** |
|  | 80 | Ex | 1.64 (0.48-5.57) | 0.428 | Ex | 1.21 (0.37-4.34) | 0.774 | |
|  |  | Current | 2.12 (0.79-5.71) | 0.138 | Current | 1.31 (0.42-4.09) | 0.647 | |
| **Lung cancer** | Total | Ex | 1.35 (1.27-1.44) | <.001 | Ex | 1.17 (1.10-1.26) | <.001 | |
|  |  | Current | 1.81 (1.74-1.88) | <.001 | Current | 1.70 (1.62-1.78) | <.001 | |
|  | 40 | Ex | 1.19 (1.01-1.39) | 0.034 | Ex | 1.09 (0.92-1.30) | 0.326 | |
|  |  | Current | 1.58 (1.43-1.74) | <.001 | Current | 1.42 (1.26-1.60) | <.001 | |
|  | 50 | Ex | 1.32 (1.16-1.51) | <.001 | Ex | 1.05 (0.91-1.20) | 0.529 | |
|  |  | Current | 2.21 (2.04-2.38) | <.001 | Current | 1.68 (1.53-1.84) | <.001 | |
|  | 60 | Ex | 1.83 (1.64-2.03) | <.001 | Ex | 1.28 (1.14-1.43) | <.001 | |
|  |  | Current | 2.78 (2.59-2.97) | <.001 | Current | 1.88 (1.74-2.03) | <.001 | |
|  | 70 | Ex | 1.88 (1.64-2.14) | <.001 | Ex | 1.24 (1.08-1.42) | 0.003 | |
|  |  | Current | 2.65 (2.42-2.90) | <.001 | Current | 1.75 (1.58-1.93) | <.001 | |
|  | 80 | Ex | 1.67 (1.08-2.58) | 0.020 | Ex | 1.27 (0.80-2.00) | 0.309 | |
|  |  | Current | 1.54 (1.03-2.31) | 0.033 | Current | 1.14 (0.75-1.74) | 0.543 | |

HR; hazard ratio, CI; confidence interval, Ex; ex-smoker, current; current smoker
