# Supplementary material for: Differential impact of smoking on cardiac or non-cardiac death according to age
Source: PLoS One. 2019 Oct 30;14(10):e0224486. doi: 10.1371/journal.pone.0224486 (PMC6821404; doi:10.1371/journal.pone.0224486)

**S1 Fig. Estimated 100,000 Person-Year Incidence rates of cardiovascular diseases according to their age and smoking habits**


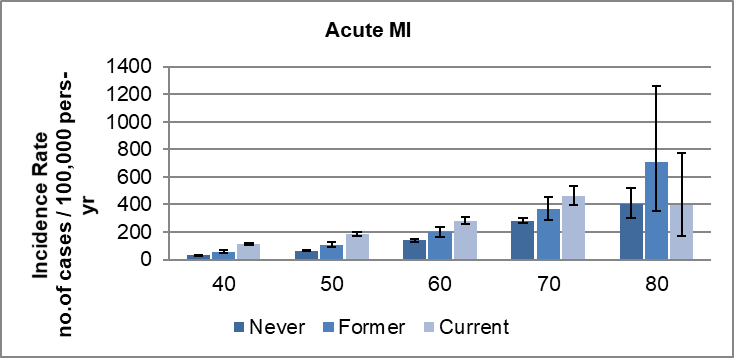

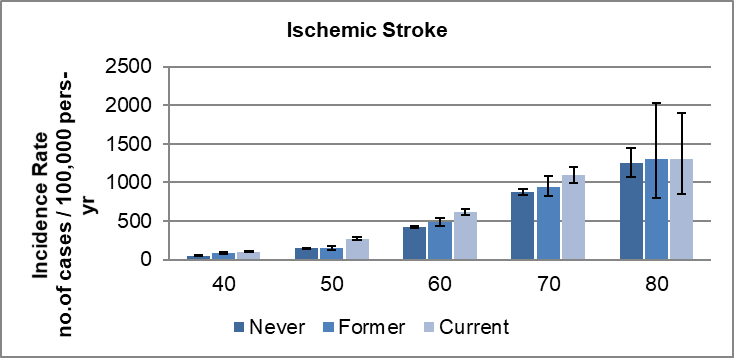

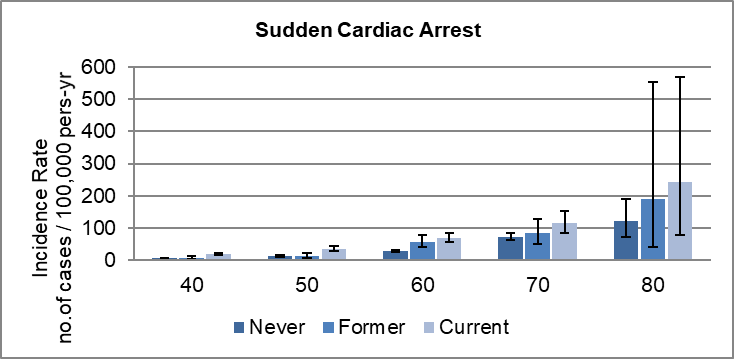

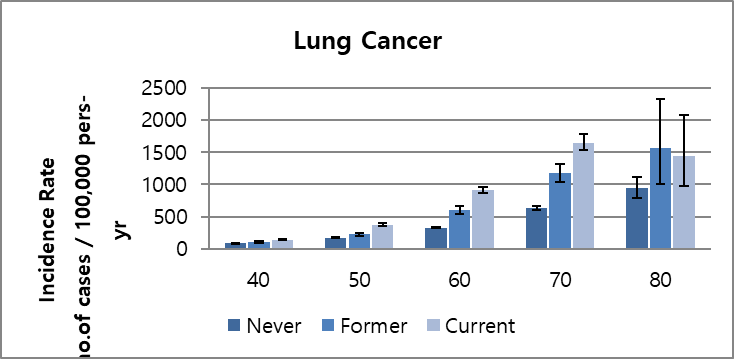

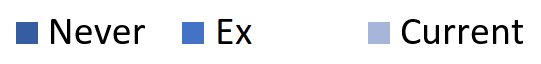

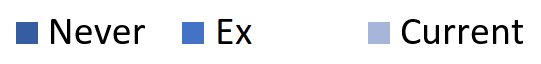

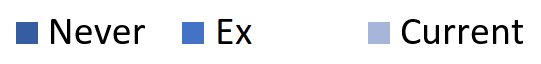

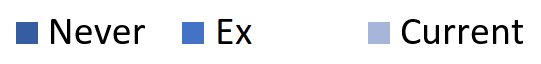

Supplement: S1 Fig — (DOCX) [file pone.0224486.s003.docx]
