# Supplementary material for: Differential impact of smoking on cardiac or non-cardiac death according to age
Source: PLoS One. 2019 Oct 30;14(10):e0224486. doi: 10.1371/journal.pone.0224486 (PMC6821404; doi:10.1371/journal.pone.0224486)

**S2 Fig. Adjusted hazard ratio and 95% confidence intervals of current smokers and ex-smokers in various outcomes**


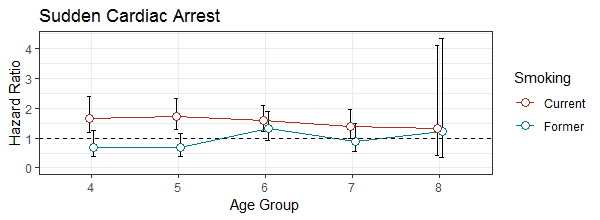

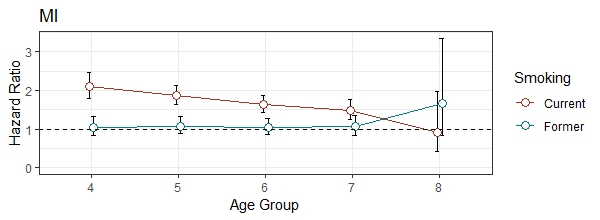

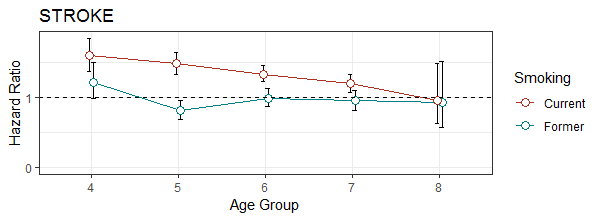

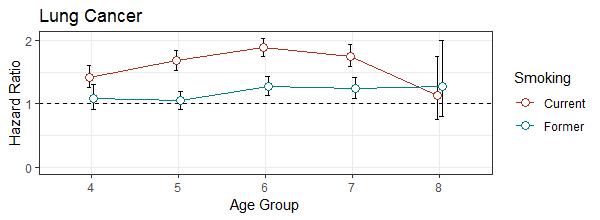

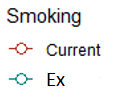

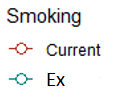

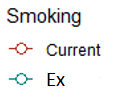

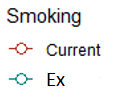

Supplement: S2 Fig — (DOCX) [file pone.0224486.s004.docx]
