# Supplementary material for: Differential impact of smoking on cardiac or non-cardiac death according to age
Source: PLoS One. 2019 Oct 30;14(10):e0224486. doi: 10.1371/journal.pone.0224486 (PMC6821404; doi:10.1371/journal.pone.0224486)
Supplement: S3 Fig — (DOCX) [file pone.0224486.s005.docx]

**S3 Fig. Proportion of current smokers according to age**
